# Propofol Inhibits Glioma Stem Cell Growth and Migration and Their Interaction with Microglia via BDNF-AS and Extracellular Vesicles

**DOI:** 10.3390/cells12151921

**Published:** 2023-07-25

**Authors:** Rephael Nizar, Simona Cazacu, Cunli Xiang, Matan Krasner, Efrat Barbiro-Michaely, Doron Gerber, Jonathan Schwartz, Iris Fried, Shira Yuval, Aharon Brodie, Gila Kazimirsky, Naama Amos, Ron Unger, Stephen Brown, Lisa Rogers, Donald H. Penning, Chaya Brodie

**Affiliations:** 1 The Mina and Everard Goodman Faculty of Life Sciences, Institute of Nanotechnology and Advanced Materials (BINA), Bar-Ilan University, Ramat-Gan 52900, Israel; rephael92@gmail.com (R.N.); matankrasner93@gmail.com (M.K.); efrat.michaely@gmail.com (E.B.-M.); doron.gerber@biu.ac.il (D.G.); jonathan.schwarz.1991@gmail.com (J.S.); gilakazimirsky@gmail.com (G.K.); amosnaama@gmail.com (N.A.); ron@biomodel.os.biu.ac.il (R.U.); 2 Davidson Laboratory of Cell Signaling and Tumorigenesis, Hermelin Brain Tumor Center, Department of Neurosurgery, Henry Ford Health, Detroit, MI 48202, USA; scazacu1@hfhs.org (S.C.); cxiang1@hfhs.org (C.X.); dpennin2@hfhs.org (D.H.P.); 3 Pediatric Hematology Oncology Unit, Shaare Zedek Hospital, Jerusalem 9103102, Israel; ishonet@gmail.com (I.F.); shira.yuval@gmail.com (S.Y.); 4 EviCure Ltd., Ness Ziona 7670306, Israel; aharon@exostem.com; 5 Radiation Oncology, Henry Ford Health, Detroit, MI 48202, USA; sbrown1@hfhs.org; 6 Department of Neurosurgery, Henry Ford Health, Detroit, MI 48202, USA; lrogers6@hfhs.org; 7 Anesthesiology, Pain Management & Perioperative Medicine, Henry Ford Health, Detroit, MI 48202, USA

**Keywords:** glioblastoma, glioma stem cells, propofol, BDNF-AS, extracellular vesicles, microglia

## Abstract

Glioblastoma (GBM) is the most common and aggressive primary brain tumor. GBM contains a small subpopulation of glioma stem cells (GSCs) that are implicated in treatment resistance, tumor infiltration, and recurrence, and are thereby considered important therapeutic targets. Recent clinical studies have suggested that the choice of general anesthetic (GA), particularly propofol, during tumor resection, affects subsequent tumor response to treatments and patient prognosis. In this study, we investigated the molecular mechanisms underlying propofol’s anti-tumor effects on GSCs and their interaction with microglia cells. Propofol exerted a dose-dependent inhibitory effect on the self-renewal, expression of mesenchymal markers, and migration of GSCs and sensitized them to both temozolomide (TMZ) and radiation. At higher concentrations, propofol induced a large degree of cell death, as demonstrated using microfluid chip technology. Propofol increased the expression of the lncRNA BDNF-AS, which acts as a tumor suppressor in GBM, and silencing of this lncRNA partially abrogated propofol’s effects. Propofol also inhibited the pro-tumorigenic GSC-microglia crosstalk via extracellular vesicles (EVs) and delivery of BDNF-AS. In conclusion, propofol exerted anti-tumor effects on GSCs, sensitized these cells to radiation and TMZ, and inhibited their pro-tumorigenic interactions with microglia via transfer of BDNF-AS by EVs.

## 1. Introduction

Glioblastoma (GBM), the most common and aggressive primary brain tumor is characterized by a high rate of proliferation, invasion into the surrounding normal tissue, robust angiogenesis, and resistance to conventional therapies [1]. The current standard of care for patients with GBM consists of tumor resection followed by a combined treatment with radiation and TMZ [2]. However, complete tumor resection is not always possible due to the infiltrative characteristics of GBM, and recurrence almost always occurs at the primary location of the tumor. Despite advances in imaging, surgical approaches, molecular tumor classification, and numerous clinical trials, the prognosis of GBM patients remains poor, with a median survival of 14–16 months, which has not significantly improved in recent decades [3].

GBM contains a small population of cancer stem cell (GSCs) that has been implicated in tumor recurrence. GSCs are characterized by their ability to self-renew, potential for multi-lineage differentiation, and high degree of invasion [4]. GSCs are resistant to conventional therapy compared with differentiated tumor cells, and recent studies indicate that both radiation and TMZ, the standard of care treatments for GBM, primarily target differentiated glioma cells [5,6]. Therefore, developing novel therapeutic approaches to target GSCs is of great importance for the treatment of GBM and for the improvement of patient prognosis.

The interaction of glioma cells and GSCs with the brain microenvironment is critical for tumor growth and treatment resistance [7]. Indeed, neural cells, such as astrocytes, neurons, and microglia, in addition to infiltrating immune cells, regulate this crosstalk [8,9]. Microglia and infiltrating macrophages represent the most prevalent GBM-associated cells [10]. These cells have been reported to undergo differentiation to an anti-inflammatory/pro-tumorigenic state in response to factors secreted by tumor cells, which further supports tumor growth [11].

One of the main components that mediate this bidirectional crosstalk are extracellular vesicles (EVs) [12]. These small nano-size vesicles play important roles in inter-cellular communication and carry diverse cargo, including proteins, lipids, and RNA molecules that mediate their effects [13,14].

The choice of general anesthetic for tumor surgical resection has been recently reported to impact tumor growth, metastasis, and recurrence, as well as prognosis of patients with solid tumors [15,16,17]. Propofol, one of the most commonly used anesthetics during surgery, has been mainly linked with anti-tumor effects [18,19]. Propofol can be used for deep general anesthesia but also in reduced dosages as a sedative, such as during awake brain surgery. It can also be used as a sedative for prolonged (days) duration for intubated patients in the intensive care unit. Retrospective epidemiological studies demonstrate that the use of propofol during resection is associated with a better prognosis than other general anesthetics [20]; however, these clinical studies are not always consistent. Anti-tumor effects of propofol in a variety of tumors, including breast, lung, and prostate were reported in a large number of in vitro and animal studies, further supporting these observations [21,22].

Here, we demonstrate that propofol exerts anti-tumor effects in GSCs and differentiated tumor cells and inhibits the tumorigenic promoting effects of microglia by the induction of BDNS-AS and its delivery via EVs.

## 2. Materials and Methods

### 2.1. Materials

Human BDNF ELISA kit was obtained from Creative Diagnostics (Shirley, NY, USA). Human TGF-β (ab178014) and IL-10 (ab185986) ELISA kits and caspase 3 activity kit (ab39401) were obtained from Abcam (Cambridge, MA, USA).

### 2.2. Experimental Protocols

Cultures were randomly viewed microscopically and assigned to the different experimental groups. All data collection and analyses were performed blinded to the treatment group. Sample sizes were determined from previous experience using these cell cultures. All data are representative of 3–6 independent experiments.

Propofol concentrations of 10 µM and 20 µM (1.8 µg/mL and 3.6 µg/mL) were employed in most studies. This is a clinically relevant concentration range, within the effect-site (loss of consciousness) concentration of 2.0–3.0 µg/mL seen in a study of human volunteers [23], and a plasma concentration of 3.6–5.7 µg/mL corresponded to a brain concentration of 2.1 to 3.2 µg/mL reported in rats [24].

All experiments were performed in medium containing EV-depleted serum, which was prepared by ultracentrifugation (100,000× *g*, 4 °C) followed by filtration of the supernatant in a 0.22 μm filter.

### 2.3. GSC and Microglia Cultures

All human materials were used in accordance with the policies of the Henry Ford Hospital Institutional Review Board.

GSCs were generated from fresh resected tumors. Pathology was graded according to the WHO criteria and IDH status. The cells were extracted, cultured as neurospheres, and characterized as previously described [25,26,27]. The GSCs were maintained in neurosphere medium (DMEM-F12 1:1, glutamine 10 mM, HEPES buffer 10 mM, and sodium bicarbonate 0.025%) supplemented with basic fibroblast growth factor and epidermal growth factor (20 ng/mL). GSCs exhibited self-renewal ability, expressed stem cell markers (Sox2, OCT4, CD133, nestin, and CD44), and generated glioma xenografts in nude mice upon transplantation [26]. The full information of the GSCs employed in this study is described in Table S1.

Differentiated cells (DGSCs) were generated by plating the GSCs in medium consisting of DMEM+10% FCS for a week. The cells lost their ability to grow as spheroids and exhibited a marked decrease in the expression of Sox2 and OCT4.

hTERT-immortalized human microglia were obtained from Applied Biological Material (Richmond, BC, Canada). The cells were maintained in growth media and conditions recommended by the manufacturer [28]. All cells were routinely tested for mycoplasma contamination (Mycoplasma PCR Detection Kit) and found negative.

### 2.4. Neurosphere Formation/Self-Renewal Assay

The ability of GSCs to form secondary neurospheres was analyzed as recently reported [25,29,30]. GSCs were dispersed into single cells and seeded at a density of 100 cells/well through limiting dilution. The number of neurospheres/well was determined following 14 days for 10 different wells. Spheres that contained more than 20 cells were scored. The results are presented as neurosphere number or percentages of maximal neurospheres formed in treated compared to control cultures.

### 2.5. In Vitro Limiting Dilution Assay

GSCs were plated in 96-well plates in decreasing numbers of cells (50, 20, 10, 5, 2, and 1) per well. After 14 days, the number of spheres was determined for each well, and extreme limiting dilution was analyzed as recently reported [30], http://bioinf.wehi.edu.au/software/elda.

### 2.6. Cell Proliferation

Cell proliferation was determined using the XTT assay according to the manufacturer’s instructions. Briefly, cells were seeded in 96-well plates (3 × 10^3^ cells/well). Following treatments, the medium was replaced with 20 μL of XTT solution, and plates were incubated for 2–4 h at 37 °C and 5% CO_2_, followed by the addition of extraction buffer. The absorbance at 590 nm was measured. Analyses were determined in quintuplets for each treatment and repeated at least 3 times.

Cell proliferation was also determined using trypan blue exclusion assay. For this assay, 3 × 10^3^ cells were plated in a 12-well culture plate and allowed to differentiate prior to propofol treatment. Control and propofol-treated cells were stained with trypan blue and total viable cells were counted using a hemocytometer. Each experiment was performed in triplicates, and the total number of viable cells and percentage of untreated cells are presented.

### 2.7. Cell Death Assays

Caspase 3 activity was analyzed using a fluorometric assay according to the manufacturer’s instructions (Abcam, ab 39383). The data were calculated as fluorescence units/mg protein and presented as fold increase over the control level.

Cell death was also analyzed using the LDH assay kit (Abcam, ab 102526) by analyzing lactate dehydrogenase (LDH) levels in culture supernatants according to the manufacturer’s instructions.

### 2.8. siRNA Transfection

GSCs in 6-well plates were transfected with control and BDNF-AS siRNA duplexes (Silencer Select, ThermoFisher Scientific, Lafayette, CO, USA) using siIMPORTER (Millipore, Billerica, MA, USA) according to the manufacturer’s instructions. Cells were collected for analysis 48 h after transfection and qRT-PCR was used to confirm BDNF-AS knockdown.

### 2.9. Lentivirus Transduction of YKL-40 Reporter

Lentivirus vectors (System Biosciences, Mountain View, CA, USA) expressing control and GFP/fLuc YKL-40 reporters were packaged and used to transduce GSCs according to the manufacturer’s protocol and as previously reported [31]. Briefly, HEK293 cells were plated at 60–70% confluency. Following 24 h, cells were transfected with a total of 15 mg/mL DNA consisting of the control and reporter plasmids and packaging plasmids pCMV-DR8.2 and the envelope plasmid VSV-G. After 18 h, the transfection mix was removed and replaced with fresh medium. Medium was collected after 48 h and centrifuged at 100,000× *g* for 90 min. Lentivirus was frozen in aliquots at −80 °C until used. GSCs were transduced with the YKL-40 or control lentivirus vectors twice with a 3 day interval, and transduction efficiency was determined by confocal microscopy and luciferase activity.

### 2.10. Luciferase Activity

The firefly luciferase activity of YKL-40 and the control Renilla luciferase were analyzed using the Dual-Luciferase^®^ Reporter Assay System (Promega Corporation, Madison, WI, USA).

### 2.11. Quantitative Real-Time PCR

qRT-PCR was performed as previously described [26,27,28]. Briefly, RNA was extracted from GSCs and microglia using miRNeasy total RNA isolation kit according to the manufacturer’s instructions (Kit #217004, Qiagen, Frederick, MD, USA). Reverse transcription reaction of 1 mg/mL was performed using superscript IV RT kit (ThermoFisher #18090010), and quantitative PCR was performed with green I dye (PowerUp™ SYBR™ Green Master Mix, Applied Biosystems) on an ABI VIIA7 Sequence Detection System (Applied Biosystems, Foster City, CA, USA). Cycle threshold (Ct) values were obtained from the ABI QuantStudio software and data were generated by comparative CT(∆∆CT) method. Housekeeping control genes (beta-2 microglobulin or ribosomal S12) were selected based on their stable expression across the relevant conditions and treatments employed in these studies.

Relative mRNA expression to S12 was calculated and data normalized to a control group. Experiments were performed in triplicates at least 3 times, and average relative expression are presented. Primer sequences are listed in Table S2.

### 2.12. EV Isolation and Characterization

EVs were isolated from culture supernatants using the ExoQuick-TC Ultra kit (SBI, Palo Alto, CA, USA) according to the manufacturer’s instructions. The protein content of the isolated EVs was determined using the Micro BCA assay kit (ThermoFisher Scientific, Oregon City, OR, USA), and their quantification was analyzed using the CD63, CD9, and CD81 ExoELISA-Ultra kits (SBI, Palo Alto, CA, USA), as recently described [27,32]. For the treatment of cultured cells, 2 × 10^8^ EVs were employed.

### 2.13. Quantification of Cytokine Secretion and EV Subpopulations

TGF-β1, IL-10, and BDNF levels were quantified in culture supernatants using enzyme-linked immunosorbent assays (ELISA) according to the manufacturer’s instructions. The concentrations of the specific cytokines were calculated using a standard curve and were expressed as picograms per milliliter.

### 2.14. Transwell Migration Assay

Cell migration was analyzed using transwell chambers with an 8 μm filter (BD Biosciences, San Jose, CA, USA), as recently reported [31,33]. Briefly, GSCs were plated in transwell chambers (25,000 cells per well) and the chambers were then incubated for 12 h in culture medium with 0.5% FBS in the bottom chambers. Cells on the upper surface were scraped, and the migrated cells on the lower surface were fixed and stained with 0.05% crystal violet for 5 min. Stained cells were counted under a microscope, and the migrating cell number was calculated.

### 2.15. Chip Manufacturing

Polydimethylsiloxane (PDMS, SYLGARD 184, Dow Corning, Midland, MI, USA) devices were manufactured using standard methods as recently described [34,35]. Briefly, the flow (cell culture) and control (valves) layers were prepared separately on silicone molds casting silicone elastomer PDMS. For the control layer, PDMS and curing agent were mixed at a 5:1 ratio, followed by degassing for 15 min, baking for an hour, and access-hole-piercing steps. The flow layer was prepared similarly, except for the application of a 20:1 ratio of PDMS to curing agent. The layers were then aligned using a home-built semi-automatic alignment system. The chip was then placed in an oven at 80 °C for full curing. Holes were punched to allow the connection of tubes via pins and the flowing of air or fluids into the chip during the experiment.

### 2.16. Chip Operation

The flow of medium/cells was regulated using a pneumatic system (regulated semi-automatically). Working pressure for cell flow was 3–5 PSI. Input and output control valves were operated with 20 PSI. The temperature, humidity, and CO_2_ were controlled by the fluorescent microscope incubator built-in system (Bold Line, Okolab, Italy).

### 2.17. Cell Viability Assays

GSCs were introduced into the main channel and the incubation chambers by horizontal flow, and cells were subjected to media flow alone or with propofol concentrations at a low pressure (3–5 PSI). The cells were stained with propidium iodide (PI) for 30 min at 37 °C.

Imaging was acquired by Nikon Eclipse Ti using NIS Elements software (ver. 4.20.01 Nikon, Melville, NY, USA) and processed with NIS Elements Analysis software (ver. 40.20.01 Nikon, USA).

### 2.18. Radiation Exposure

The radiation source used in this study was a Cesium (Cs-137) irradiator (Mark I Model 68, J.L. Shepherd and Associates, San Fernando, CA, USA; serial number: 1048). The source’s activity was 5000 Ci (J.L. Shepherd and Associates Type 6810; serial number: 87CS-S-30) at the time of installation, 24 June 1988, and approximately half that during the study duration. The radiation dose was mapped by Shepherd and Associates in 1988 and confirmed in 2006 (Model 2025 X-ray monitor with a 0.18 cc chamber, MDH Industries Inc., Monrovia, CA, USA). Independent measurements of radiation dose were made prior to the study using a small-sized cylindrical ion chamber (PTW PinPoint Ion Chamber Type 31014, Freiburg, Germany), which was 2 mm in diameter and had a sensitive volume of 0.015 cm^3^, and radiographic film (EBT3 Gafchromic film, Ashland, Bridgewater, NJ, USA). The electrometer measurements were used to confirm that the radiation doses provided by the manufacturer, JL Shepherd, were within ±3%. The radiation dose rate during the course of the experiments was approximately 1 Gy/min. The radiographic x-ray film measurements were used to demonstrate uniformity of radiation field. Film placed under the cells confirmed that exposures were uniform (±3%) following an exposure with a rotating platform.

### 2.19. TCGA Data Analysis

Gene expression data for adult LGG and GBM patients were downloaded from the GDC Data Portal (Release Date 2022-03-29: https://portal.gdc.cancer.gov/). Sequencing was performed using Illumina technology, and alignment was carried out using the “STAR 2-Pass Genome” workflow. Expression levels of BDNF and BDNF-AS normalized by TPM (Transcripts Per Million) were extracted from these files. The corresponding patient clinical data were taken from a study of molecular profiling of glioma subtypes [36].

BDNF-AS and BDNF levels were analyzed in different subtypes of LGG and GBM and in relation to tumor recurrence and patient survival. Expression data are presented graphically with median and interquartile range noted. Comparison of mean expression between groups was performed by one-way ANOVA followed by Tukey’s corrected two-sample tests, which adjust for multiple comparisons to maintain the family-wise error rate. Kaplan–Meier estimates of the survival time from diagnosis until death or last follow-up were used for outcome analysis. Differences in survival curves between groups were assessed by the log-rank test. Cox regression was used to construct multivariable models of survival, including mRNA expression, age at diagnosis, IDH mutation status, and grade. Graphs were generated using R (v.4.2.3).

### 2.20. Statistical Analysis

All experiments were repeated at least 3 times. Statistical significance was determined using two-tailed unpaired Student’s *t*-test for the comparison of two groups or with one-way ANOVA with Tukey’s test for experiments with more than two groups. Data were analyzed using ANOVA or a Student’s *t*-test with correction for data sets with unequal variances. A two-way ANOVA was conducted to examine the interaction between two treatments. Error bars represent mean ± SD unless otherwise stated. *p*-value of < 0.05 was considered statistically significant.

## 3. Results

### 3.1. Propofol Inhibits the Self-Renewal and Stemness of GSCs and Proliferation of Differentiated Glioma Cells

Propofol has been reported to exert anti-tumor effects on various established glioma cell lines [37,38,39]; however, its effects on GBM-tumor-patient-derived primary cultures are not reported. We chose to employ patient-tumor-derived glioma stem cells (GSCs) in this study, since they play key roles in glioma initiation, progression, and resistance to existing therapies. Studying these cells allows analysis of both cancer stem cells and their differentiated glioma-cell (DGSCs) progeny. Importantly, GSCs have been also demonstrated to generate reliable GBM models that recapitulate the characteristics of the parental tumors in vitro and in vivo [39].

**Figure 1:**
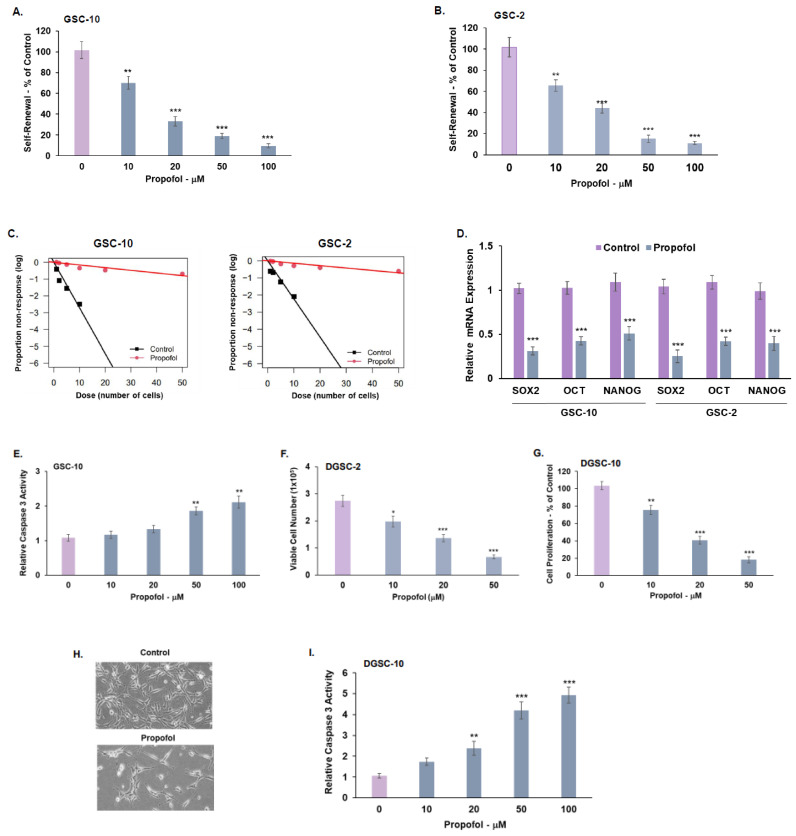

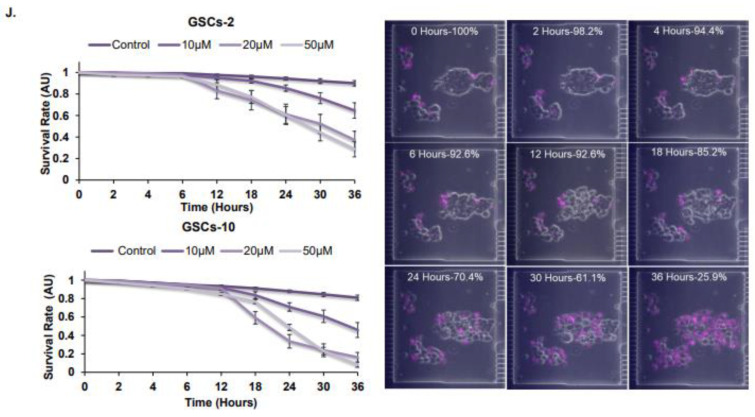
Propofol inhibits the self-renewal and stemness in GSCs and cell proliferation in DGSCs. GSC-10 (**A**) and GSC-2 (**B**) were treated with different concentrations of propofol. For self-renewal analysis, GSCs were plated at 100 cells/well in 96-well plates, and the number of neurospheres per well was quantified after 14 days. Self-renewal is determined as % of normalized control cells (**A**,**B**). In vitro extreme limiting dilution assay (ELDA) was performed in control and propofol-treated (20 μM) GSC-10 and GSC-2 (**C**). The expression of stemness markers was determined using RT-PCR (**D**). Cell apoptosis of propofol-treated GSC-10 was analyzed using caspase 3 activity (**E**). GSC-10 were differentiated by plating in serum-containing medium for a week. DGSCs were then treated with various propofol concentrations. Cell proliferation was determined by determining cell number via trypan blue exclusion assay (**F**) and XTT assay (**G**). A representative image of the control and propofol-treated (20 μM) differentiated GSCs (DGSCs) is presented (**H**). Cell apoptosis of DGSC-10 was determined using caspase 3 assay (**I**). GSC-10 and GSC-2 were plated on a microfluid chip and treated with different propofol concentrations, and cell death was monitored over 36 h using propidium iodide staining. The graphs present the survival rates of the treated cells by subtracting the normalized survival rate (100%) at 0 h time point from the survival rate at each treatment time point. Images demonstrate survival rate using PI staining of propofol-treated cells (20 μM) at different time points. *p* < 0.001 (**J**). The results are presented as the means ± SD of four different experiments. * *p* < 0.05, ** *p* < 0.01, *** *p* < 0.001. Significance was determined by two-tailed unpaired Student’s *t*-test.

Using tumor-derived GSCs (Table S1), we first analyzed the effects of propofol on the self-renewal of these cells. GSCs were treated with different concentrations of propofol, and the generation of secondary neurospheres was determined 14 days thereafter. Propofol exerted a dose-response inhibitory effect on the self-renewal of GSC-10 and GSC-2 (Figure 1A,B), starting at a concentration of 10 μM. Similar results were obtained using extreme limiting dilution assay (Figure 1C). In addition, propofol also decreased the expression of the stemness markers SOX2, OCT4, and Nanog in both GSC-10 and GSC-2 (Figure 1D), further indicating that propofol inhibited the stemness potential of these cells.

Propofol effects were also observed in two additional GSC lines (Figure S1A,B). The inhibitory effects of propofol on the self-renewal of GSCs at concentrations of 10–20 μM were not attributed to increased cell death, since propofol induced cell death in GSCs only at higher concentrations of 50–100 μM (Figure 1E and Figure S1C–E).

GSCs represent a small percentage of the cellular components of GBM, whereas most of the tumor is comprised of differentiated tumor cells. We therefore also analyzed the anti-tumorigenic effects of propofol in GSC-derived differentiated tumor cell progeny (DGSCs). Propofol inhibited cell proliferation in a dose-dependent manner, starting at a concentration of 10 μM, as indicated by cell counting (Figure 1F), XTT analysis (Figure 1G), and images of cell confluency (Figure 1H). Propofol also induced apoptotic cell death in the DGSCs, starting at a concentration of 20 μM (Figure 1I).

We further analyzed propofol’s effects, also using a microfluidic platform that allowed the monitoring of cell survival in living cells. The GSC spheroids were dissociated, cultured in chip chambers, treated with various concentrations of propofol, and continuously imaged by fluorescent microscopy. As presented in Figure 1J, under these conditions, propofol induced a large degree of cell death, and cell survival decreased to 20–30% at 36 h in both GSC-10 and GSC-2, as demonstrated both graphically and by fluorescence imaging of PI labelled cells.

Different anesthetics have been reported to exert short and long-term neural cytotoxic effects [40]. We therefore analyzed the effects of propofol on human neural cells. Importantly, propofol at a concentration of 100 μM did not induce a significant degree of cell apoptosis in human neurons, microglia, or oligodendrocytes (Figure S2A), or cytotoxic effects in neurons, astrocytes, microglia, and oligodendrocytes (Figure S2B). These results indicate that propofol effects on tumor cells are selective and are not accompanied by neural cell toxicity.

### 3.2. Propofol Inhibits the Migration of GSCs and Their Mesenchymal Transit

**Figure 2:**
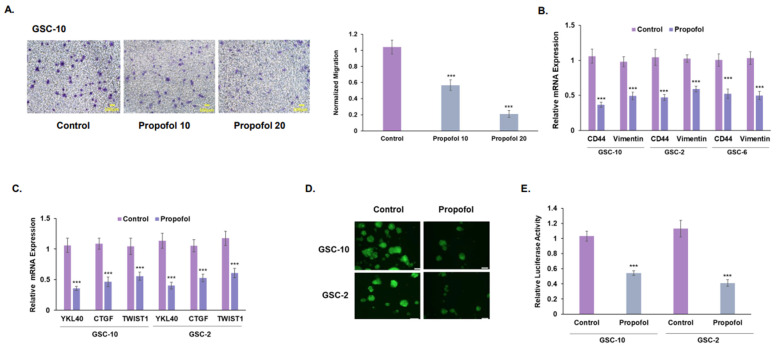
Propofol inhibits GSC migration and mesenchymal transit. GSCs were treated with propofol (20 μM) for 24 h. Cell migration was analyzed using transwell migration assay. Experiments were performed in triplicates and repeated three times. Five fields were analyzed for each well (**A**). The expression of mesenchymal markers CD44 and vimentin (**B**) and that of YKL40, CTGF, and TTWIST1(**C**) was analyzed using qRT-PCR. GSCs were transduced with lentivirus vectors expressing a control and GFP/fLuc-YKL-40 reporter and treated with propofol (20 μM) for 48 h. Cell fluorescence was imaged using fluorescent microscopy (**D**), and luciferase activity was determined (**E**). The results represent the means ± SD of three different experiments analyzed in quadruplets. *** *p* < 0.001. Significance was determined by two-tailed unpaired Student’s *t*-test.

Glioma cells exhibit a high degree of migration, which is usually associated with mesenchymal phenotypes [41]. We found that propofol decreased the migration of GSCs (Figure 2A) and DGSC-10 (Figure S3A). In addition, propofol also inhibited the mesenchymal transit of GSCs, as demonstrated by the decreased expression of the mesenchymal markers CD44 and vimentin (Figure 2B) and that of CTGF, TWIST1, and YKL-40 (Figure 2C). We further analyzed propofol’s effect on the mesenchymal transit of GSCs using a YKL-40 reporter. GSCs were transduced with lentivirus vectors expressing YKL-40 promoter tagged to GFP/fLuc or a control GFP/fLuc empty vector, treated with propofol and analyzed for GFP fluorescence and luciferase activity. As with the RT-PCR analysis results, we found that propofol decreased YKL-40 expression in the GSCs, as indicated by the decreased fluorescence (Figure 2D) and luciferase activity (Figure 2E) of the treated cells. Altogether, these results demonstrate that propofol acts as a negative regulator of the mesenchymal phenotype and migration of GSCs.

### 3.3. Combined Effects of Propofol, TMZ, and Radiation in GSCs and DGSCs

**Figure 3:**
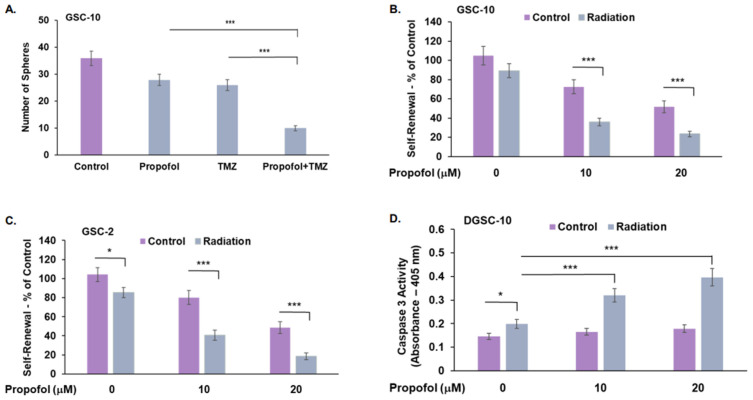
Combined effects of propofol, TMZ, and radiation. GSCs were treated with propofol alone (10 μM) or in the presence of TMZ (25 μM) or radiation (1 Gy). Self-renewal results of GSC-10 treated with TMZ and propofol are represented by the total number of spheres. The combined treatment exerted an additive effect. Interaction analysis using two-way ANOVA demonstrated that there was no significant interaction between propofol and TMZ, indicating an additive effect; F(1, 16) = 2.076, *p* = 0.169 (**A**). GSC-10 (**B**) and GSC-2 (**C**) were treated with propofol and radiation and the generation of secondary spheroids was analyzed after 14 days. Results are presented as the means ± SD of four different experiments (**B**,**C**). Cell death of GSC-10 treated with propofol and radiation (1 Gy) was determined after 24 h by analyzing caspase 3 activity (**D**). The results represent the means ± SD of five different experiments, and the significance between the different treatments was determined using one-way ANOVA with Tukey’s multiple comparison test * *p* < 0.05, *** *p* < 0.001. Interaction between the different treatments was analyzed by two-way ANOVA. Other than the combined effects of TMZ and propofol on the self-renewal of GSCs (**A**), all other combined treatments resulted in statistically significant interactions, indicating a synergistic effect *p* < 0.008.

TMZ and radiation are the standard treatments of care for GBM patients following surgery [42]. We therefore examined the effects of propofol on the response of GSCs to TMZ and radiation. We found that propofol enhanced the inhibitory effect of TMZ (25 μM) on the self-renewal of GSC-10 (Figure 3A) in an additive manner, whereas a synergistic effect was observed on cell proliferation of DGSC-2 (Figure S3B).

Similar results were obtained for combined treatments of propofol and radiation. We employed a dose of 1 Gy radiation, since the aim of this experiment was to analyze the ability of propofol to sensitize GSCs to radiation, and this dose induced a small number of cytotoxic effects in these cells. Irradiation of the cells exerted a small inhibitory effect on the self-renewal of GSC-10 and GSC-2. Combined treatment of GSCs with radiation and propofol (10 μM and 20 μM) exerted synergistic effects on the self-renewal of GSCs (Figure 3B,C) and apoptosis of DGSCs (Figure 3D).

### 3.4. Propofol Increases the Expression of the lncRNA BDNF-AS in GSCs

Propofol exerts anti-tumor effects via diverse mechanisms, including changes in the expression of specific non-coding RNAs [43]. We analyzed the effects of propofol on the expression of the lncRNA BDNF-AS. This lncRNA is transcribed from the opposite strand of BDNF and therefore acts as a negative regulator of BDNF expression [44]. BDNF has been reported to play a role in the tumorigenesis of glioma and oncogenic potential of GSCs [45], whereas recent studies have reported that BDNF-AS acts as a tumor suppressor in glioma [46].

**Figure 4:**
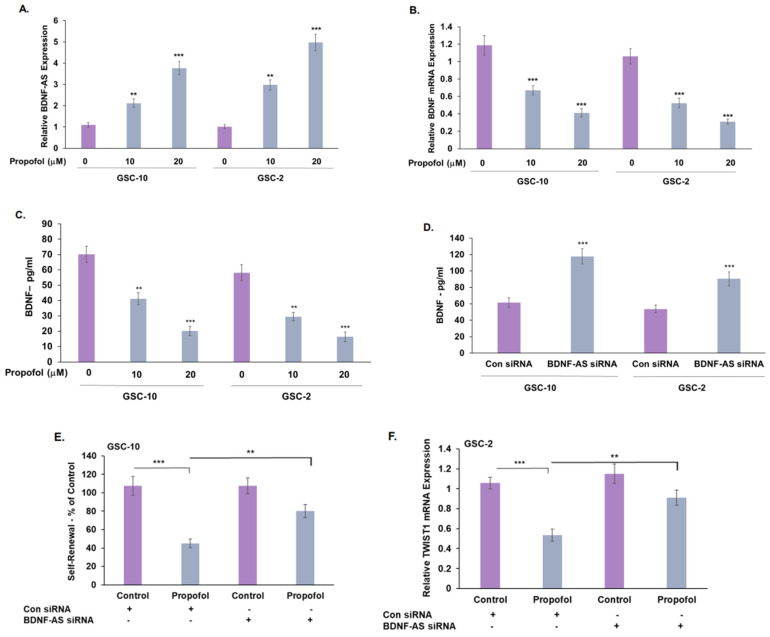
Expression and role of BDNF-AS in propofol’s effects. The effects of propofol (20 μM) on BDNF-AS (**A**) and BDNF (**B**) expression was determined in GSC-10 and GSC-2 cells after 24 h of treatment using RT-PCR. BDNF secretion in propofol-treated GSCs was determined by ELISA (**C**). The role of BDNF-AS in propofol effects was analyzed in GSCs that were silenced for the expression of BDNF-AS. GSCs were transfected with a BDNF-AS siRNA for 24 h, followed by treatment with propofol for additional 24 h. The secretion of BDNF was analyzed using ELISA (**D**) and propofol effects on GSC-10 self-renewal after 14 days (**E**), and TWIST1 expression in GSC-2 after 48 h (**F**) was determined. The results represent mean values ± SD of four different experiments. ** *p* < 0.01, *** *p* < 0.001. Significance was determined by two-tailed unpaired Student’s *t*-test.

Treatment of GSCs with propofol increased the expression of the lncRNA BDNF-AS (Figure 4A). In contrast, this treatment decreased the expression (Figure 4B) and secretion of BDNF (Figure 4C).

To delineate the role of BDNF-AS in propofol’s effects, we silenced the expression of BDNF-AS in the GSCs and analyzed their self-renewal, stemness, and mesenchymal marker expression as well as BDNF-secretion. Transfection of GSC-10 with two BDNF-AS siRNA decreased the expression of this lncRNA by around 80% (Figure S4). BDNF-AS silencing increased BDNF secretion in GSCs (Figure 4D) and partially abrogated the inhibitory effect of propofol on GSC self-renewal (Figure 4E) and TWIST1 expression (Figure 4F).

**Figure 5:**
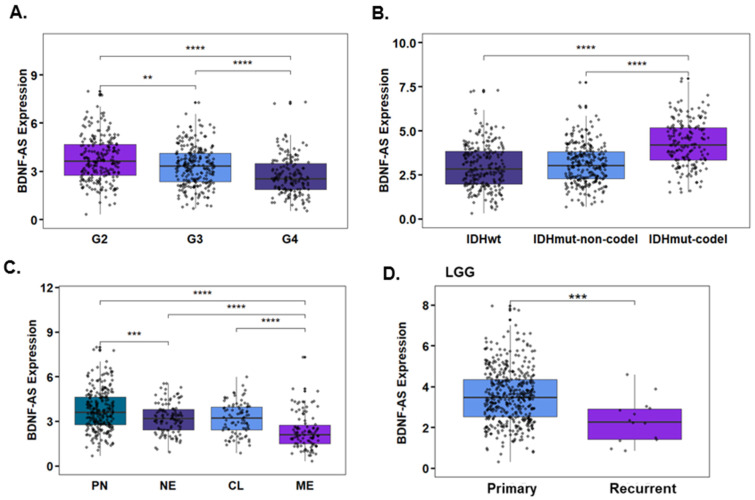

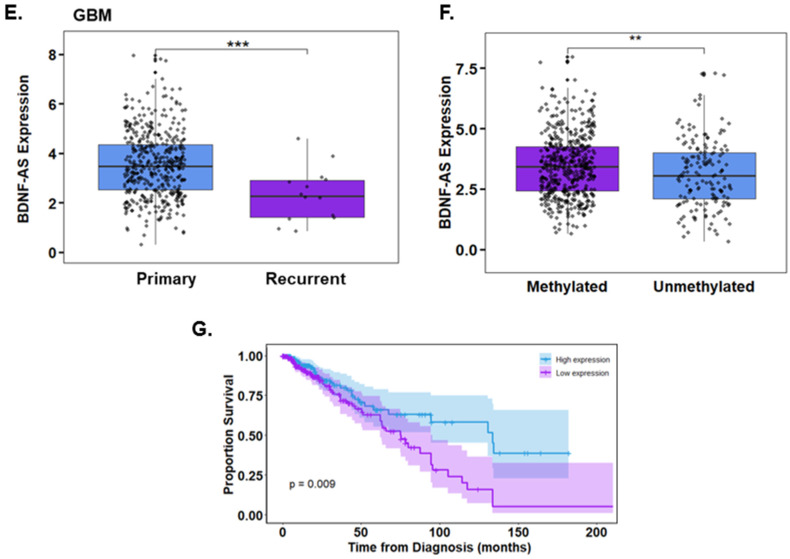
Expression of BDNF-AS in glial tumors. Relative expression of BDNF-AS in LGG (G2, *n* = 220; G3. *n* = 241) and GBM (G4, *n* = 162) was determined according to The Cancer Genome Atlas (TCGA data portal, Wilcoxon *t*-test *p* < 0.0001) (**A**). BDNF-AS expression was also determined in glioma tumors (GBM and LGG) by IDH status (IDH WT, *n* = 232; IDHmut-codel *n* = 171; IDHmut-non-codel, *n* = 268, Wilcoxon *t*-test *p* < 0.0001) (**B**), and molecular subtypes (PN, *n* = 250; NE, *n* = 109; CL, *n* = 88l; ME, *n* = 102, Wilcoxon *t*-test *p* < 0.001) (**C**). The expression of BDNF-AS was analyzed in primary (*n* = 444) and recurrent (*n* = 14) LGG tumors, *p* < 0.0001 (**D**), primary (*n* = 140) and recurrent (*n* = 13) GBM tumors (**E**), and in MGMT methylated (*n* = 489) and unmethylated (*n* = 160) tumors (LGG+GBM), *p* < 0.01 (**F**). Overall survival of LGG patients was determined using a Kaplan–Meier survival model, and the *p*-value was calculated using log-rank test, *p* = 0.009 (**G**). ** *p* < 0.01, *** *p* < 0.001, **** *p* < 0.0001.

We then performed a bioinformatic analysis of BDNF-AS expression in glioma using a TCGA dataset. A total of 162 cases of GBM and 461 cases of LGG were analyzed. The expression of BDNF-AS was decreased in GBM (G4) compared to LGG (G2, G3) tumors (*p* < 0.0001, Figure 5A). Similarly, we found that the expression of BDNF-AS was significantly downregulated in IDHwt compared with IDHmutant tumors (*p* < 0.001, Figure 5B) and in mesenchymal compared with proneural subtypes (*p* < 0.0001, Figure 5C), indicating that aggressive tumors with poor prognosis are associated with lower levels of BDNF-AS. Analyzing the expression of BDNF-AS in primary and recurrent LGG (Figure 5D) and GBM (Figure 5E) tumors demonstrated a significant decrease in both types of recurrent tumors (*p* < 0.01 for GBM and *p* < 0.001 for LGG). BDNF-AS expression was also increased in MGMT methylated compared with unmethylated glioma tumors (GBM and LGG) (Figure 5F), and this increased expression was observed in both primary (Figure S5A) and recurrent tumors (Figure S5B). No significant differences in BDNF-AS expression were observed when GBM (Figure S5C) and LGG (Figure S5D) were analyzed separately. Finally, we also found that higher levels of BDNF-AS are associated with increased overall survival in LGG patients (Figure 5G), whereas no significant association was observed in patients with GBM (Figure S5E).

The expression of BDNF displayed an opposite pattern and was higher in GBM compared with LGG (Figure S5F), in IDHwt compared with IDH mutant tumors (Figure S5G), and in mesenchymal compared with proneural tumors (Figure S5H). BDNF expression was significantly increased in recurrent GBM (Figure S5J, *p* < 0.05) but not in recurrent LGG tumors (Figure S5I). BDNF expression was not associated with changes in patient overall survival in either GBM (Figure S5K) or LGG (Figure S5L) patients, suggesting that BDNF may not be the only target of BDNF-AS.

### 3.5. Treatment of GSCs with Propofol Decreases the Anti-Inflammatory/Pro-Tumorigenic State of Co-Cultured Microglia via the Transfer of EVs

We then examined whether propofol also affected the interaction of GSCs with microglia cells in addition to its direct effect on the tumor cells.

**Figure 6:**
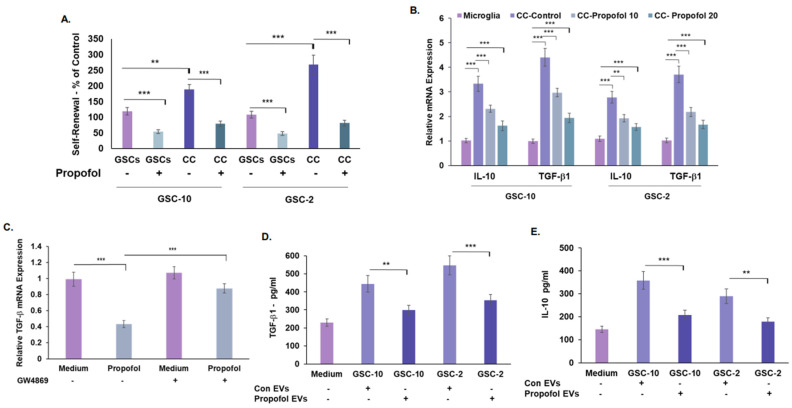
Propofol induces pro-inflammatory/anti-tumorigenic phenotypes of co-cultured human microglia via EVs. GSCs and microglial cells were cultured alone or in co-cultures in transwell plates with 1-μm filters (CC). Self-renewal of co-cultured GSCs (CC) was analyzed after 14 days, as described in Figure 1A (**A**). The expression of IL-10 and TGF-β1 (anti-inflammatory M2-like markers) in microglia plated alone and in co-culture (CC) was determined by RT-PCR (**B**). GSC-10 were treated with GW4869 for 20 h followed by propofol treatment for additional 24 h. The cells were then co-cultured with microglia in transwell plates for 24 h. The expression of TGF-β1 in the microglia cells was determined by RT-PCR (**C**). GSCs were treated with propofol (20 μM) for 24 h and EVs were isolated using ExoQuick TC Ultra kit. Microglia were treated with EVs (2 × 10^8^/mL) isolated from untreated (Medium) or propofol-treated cells (20 μM) for 24 h, and the secretion of TGF-β1 (**D**) and IL-10 (**E**) was determined by ELISA. The results represent mean values ± SD of four different experiments. Significance was determined using one-way ANOVA with Tukey’s multiple comparison test ** *p* < 0.01, *** *p* < 0.001.

Recent studies have demonstrated a bidirectional crosstalk of glioma cells and microglia, which promotes tumor growth [46]. We analyzed the effects of propofol on microglia state and GSCs functions in transwell co-culture plates with 1 μm filters that allowed only the transfer of soluble factors. As we recently reported [26], co-culturing of GSCs with microglia cells (CC) increased the self-renewal of the GSCs (Figure 6A). Under these culture conditions, the relative expression of the anti-inflammatory/pro-tumorigenic cytokines, TGF-β1, and IL-10 was increased in the co-cultured microglia cells [47] (Figure 6B). Treatment of the co-cultured GSCs with propofol (10 and 20 μM) decreased the self-renewal of these cells (Figure 6A) and the expression of M2-like microglia cytokines in the co-cultured microglia cells (Figure 6B).

We and others recently reported that EVs play an important role in the crosstalk of glioma cells and microglia [26]. We therefore analyzed the role of EVs in propofol effects on this intercellular interaction. To analyze the roles of EVs in propofol’s effects on GSC-microglia interactions, we first employed the membrane neutral sphingomyelinase (nSMase) inhibitor, GW4869, which reduces the secretion of EVs by blocking the ceramide-dependent budding of intraluminal vesicles (ILV) into the lumen of multivesicular bodies (MVBs) [48]. GSC-10 were treated with GW4869 (20 μM) prior to propofol treatment and their co-culture with microglia. GW4869 treatment decreased EV secretion in GSC-10, as demonstrated by CD63 ELISA (Figure S6A), and partially abrogated the inhibitory effect of propofol on the microglial expression of TGF-β1 Figure 6C.

To further analyze the role of EVs in propofol’s effect, we isolated EVs from control and propofol-treated GSCs and examined their effects on microglia, as previously reported [26]. We analyzed the EVs isolated from both control and propofol-treated GSCs for CD63-, CD81-, and CD9-positive EV subpopulations by ELISA. We found that treatment of GSCs with propofol did not induce a significant change in the amount of the CD81+ and CD63+ EVs, whereas a small decrease was observed in the amount of CD9+ EVs (Figure S6B).

We then examined the effects of EVs isolated from control and propofol-treated GSCs on microglia cells. EVs isolated from control GSC-10 and GSC-2 increased the expression of TGF-β1 (Figure 6D) and IL-10 (Figure 6E) in microglia cells, as recently reported [26]. In contrast, EVs isolated from propofol-treated GSCs abrogated these effects, and the levels of TGF-β1 and IL-10 were significantly lower and similar to that of untreated microglia cells (Figure 6D,E).

Altogether, these results indicate that EVs secreted from propofol-treated GSCs play at least a partial inhibitory role in the interaction of GSCs with microglia cells.

### 3.6. Propofol Effects on Co-Cultured Microglia State Are Mediated by Transfer of EV-Associated BDNF-AS

**Figure 7:**
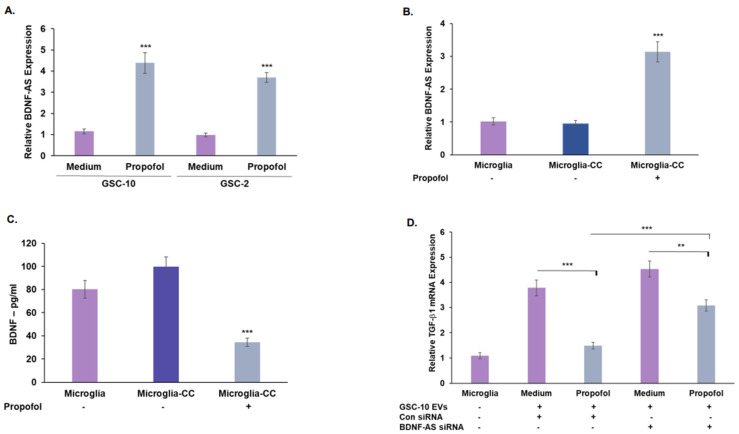
Propofol inhibits the GSC-induced pro-tumorigenic state of microglia by the delivery of EV-associated BDNF-AS. The expression of BDNF-AS was determined in EVs isolated from GSCs treated with propofol (20 μM) for 24 h as compared with control untreated cells. *** *p* < 0.001, Student *t*-test (**A**) and in microglia cultured alone or co-cultured with GSC-10 (Microglia-CC) untreated or treated with propofol (20 μM) for 24 h. *** *p* < 0.001. (**B**). The secretion of BDNF in microglia co-cultured with control or propofol-treated GSC-10 was determined by ELISA. *** *p* < 0.001, Student *t*-test. (**C**). To analyze the of role of EV-derived BDNF-AS in the effect of propofol on the GSC-microglia crosstalk, GSC-10 were transfected with a control or BDNF-AS siRNAs using siIMPORTER. After 24 h, the cells were treated with propofol for an additional 24 h, and EVs were isolated and administered to microglia cultures. The expression of TGF-β was determined in microglia cells or in microglia treated with EV-isolated from control GSC-10, GSC-10 treated with propofol, and GSC-10 transfected with BDNF-AS siRNA untreated or treated with propofol. ** *p* < 0.01, *** *p* < 0.001 (**D**). (**B**–**D**) The results represent mean values ± SD of four different experiments. Significance was determined by ANOVA with Tukey’s test.

We recently demonstrated that BDNF-AS is expressed in EVs and can modulate microglia polarization state [28]. We therefore analyzed the expression of BDNF-AS in secreted EVs and its role in abrogating the induction of anti-inflammatory/pro-tumorigenic microglia induced by co-cultured GSCs. Propofol increased the expression of BDNF-AS in EVs isolated from treated GSCs compared with untreated cells (Figure 7A). We also found that microglia co-cultured with propofol-treated GSCs exhibited an increased expression of BDNF-AS (Figure 7B) and decreased BDNF expression (Figure 7C) compared with microglia cultured with untreated cells.

We then analyzed the role of EV-associated BDNF-AS in propofol’s effect in the crosstalk of GSCs and microglia. Microglia were treated with EVs isolated from GSCs that were silenced for BDNF-AS and were either treated or untreated with propofol. We found that silencing of BDNF-AS in control GSCs did not affect the ability of secreted EVs to induce polarization of microglia to an anti-inflammatory/pro-tumorigenic state. However, BDNF-AS silencing abrogated the inhibitory effect of EVs from propofol-treated GSCs on the anti-inflammatory/pro-tumorigenic microglia polarization (Figure 7D). Thus, we conclude that the increased expression of BDNF-AS mediates both the direct anti-tumor effects of propofol on GSCs as well as the indirect effects of inhibiting the pro-tumorigenic crosstalk of GSCs with microglia via the transfer of BDNF-AS by EVs.

## 4. Discussion

GBM is one of the most aggressive, infiltrative and incurable tumors, with an average patient survival of around 14–16 months [1,2]. GBM therapy resistance and recurrence are primarily attributed to the existence of GSCs [48,49,50] and mesenchymal transition of these tumors, which is associated with the acquisition of stemness characteristics and tumor aggressiveness [51,52,53,54]. Therefore, targeting these cells and processes are an essential component of a successful therapy.

The choice of general anesthetic for tumor surgical resection has been recently reported to impact tumor growth, metastasis, and recurrence, as well as prognosis of patients with solid tumors [18,19,22]. Propofol, one of the most commonly used anesthetics during surgery, has been mainly associated with anti-tumor effects, while few studies report an opposite effect [19,22,55]. Retrospective epidemiological studies demonstrate that the use of propofol during resection is associated with a better prognosis than other general anesthetics in patients with different solid tumors [18,20]. The results of the clinical studies are not always consistent due to the retrospective nature of most reports, the wide spectrum of tumor presentation at surgery and the relatively brief single exposure of the patients to propofol. Propofol can be used for deep general anesthesia, but also in reduced dosages as a sedative, such as during awake brain surgery. It can also be used as a sedative for prolonged (days) duration for intubated patients in the intensive care unit. Longer or repetitive exposures to propofol at sedative dosages may be beneficial as an anti-tumor treatment. With regards to glioma, results of clinical studies of propofol effects during craniotomy are inconsistent [20], and there are no randomized clinical trials in neurosurgical patients on the effects of anesthetic choice and patient outcome.

More consistent effects have been reported on in vitro and animal studies, which demonstrated anti-tumor effects of propofol in a variety of solid tumors [21,22,55], further supporting these observations. Similarly, anti-tumor effects of propofol were reported on different glioma cell lines both in vitro and in vivo, mostly demonstrating changes in cell proliferation, migration [56,57], and inhibition of tumor growth [57,58]. However, all these studies were performed in specific established cell lines, whereas propofol effects on tumor-derived primary cultures of glioma cells and glioma stem cells and on the interaction of glioma cells and microglia cells are not yet reported.

Using patient-tumor-derived GSCs, we found that propofol inhibited the self-renewal and generation of secondary spheres and decreased their migration and mesenchymal transition, while inducing cell death at high concentrations. Propofol inhibited cell growth and migration also in differentiated tumor cells (DGSCs) further indicating that propofol exerts anti-tumor effects on both cell types that constitute GBM tumors. Importantly, propofol did not affect the survival of neural cells in these concentrations, highlighting the safety of this treatment. This last point is supported by the long safety record of propofol in clinical anesthesia practice.

Propofol also enhanced the cytotoxic effects of TMZ and radiation in GSCs and DGSCs, indicating that it can be combined with and may sensitize GBM to the treatments of care of these tumors. Therefore, propofol may exert significant anti-tumor effects, not only as the general anesthetic of choice during tumor resection, but also as an adjunct for current GBM treatments.

Propofol induces anti-tumor effects via multiple mechanisms [27,57,58,59], including changes in the expression of specific lncRNAs. We found that propofol induced the expression of the lncRNA BDNF-AS in GSCs, and that silencing of this lncRNA abrogated the anti-tumor effects of propofol, indicating that the increase in BDNF-AS by propofol mediated at least some of its effects.

BDNF-AS is downregulated in a variety of tumors and acts mainly as a tumor suppressor [58]. Using TCGA analysis, we demonstrated that BDNF-AS is downregulated in GBM and in glioma subtypes that are associated with aggressive phenotypes and poor prognosis, whereas high levels of BDNF-AS are associated with better patient prognosis. In addition, decreased BDNF-AS expression was also observed in recurrent LGG and GBM and in MGMT unmethylated tumors. This lncRNA is transcribed from the opposite strand of BDNF and therefore acts as a negative regulator of BDNF expression [46,60]. Propofol decreased the expression and secretion of BDNF from GSCs, and silencing of BDNF-AS increased its expression. Therefore, one mechanism of the anti-tumor effect of propofol may be mediated by decreasing BDNF levels in GSCs.

Indeed, BDNF has been reported to act as an oncogenic protein in various types of tumors, including glioma. The oncogenic effects of BDNF are mediated by binding to TrkB and the crosstalk of this receptor with the EGF receptor [61]. In addition, BDNF has been implicated as an important paracrine factor in the growth of GSCs via crosstalk of these cells with DGSCs [62].

Additional mechanisms involved in the anti-tumor effects of BDNF-AS include epigenetic suppression of GSK-3β expression in colorectal cells [62], targeting of miR-214 and EMT in esophageal cancer cells [63], stabilization of BDNF-AS in glioma cells by PABPC1 and STAU1-mediated decay [46], and stabilization of p53 in glioma cells via a positive feedback loop of BDNF-AS-ADAR-p53 [64].

Glioma cells and GSCs interact with microglia cells, and this interaction, which is partly mediated by EVs, results in the differentiation of microglia towards anti-inflammatory/pro-tumorigenic states, which promotes growth and mesenchymal transition of glioma cells [41]. Indeed, we recently demonstrated that co-culture of GSCs promoted the anti-inflammatory/pro-tumorigenic state of co-cultured microglia [26]. Using a similar transwell of co-cultures of GSCs and human microglia, we found that propofol abrogated this interaction, inhibiting the increased stemness of GSCs and the expression of TGF-β and IL-10 in the co-cultured microglia cells. Indeed, TGF-β has been reported to maintain the tumorigenicity of GSCs [65]. In addition, we demonstrated that the effects of propofol-treated GSCs on cultured microglia cells were mediated by EVs via the delivery of BDNF-AS.

The molecular mechanisms of the EV-derived BDNF-AS effects on microglia functions are currently not understood; however, recent studies have demonstrated that microglia secrete BDNF, and that this factor plays a role in microglia and macrophage activation. Repression of GSK-3β expression by BDNF-AS can also contribute to the promotion of microglia M1-like polarization.

**Figure 8:**
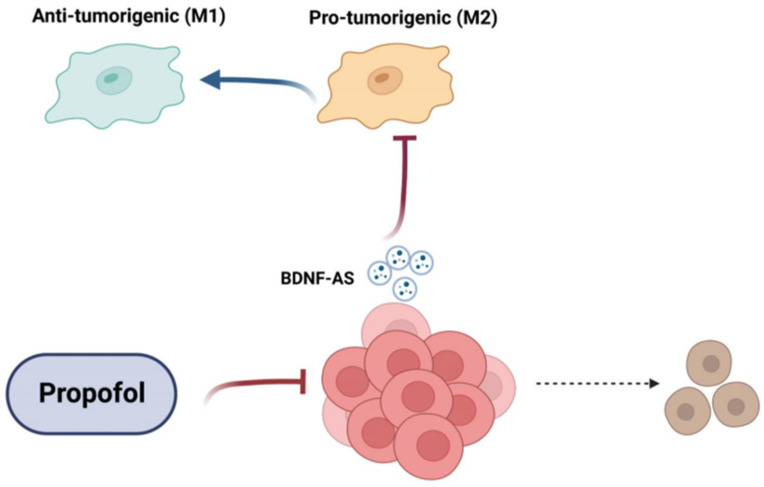
A diagram summarizing the effects of propofol on GSCs and their crosstalk with microglia. The anti-tumor effects of propofol on GSCs, their crosstalk with microglia, and the role of EVs in this interaction are depicted (created with BioRender.com). PMCID: PMC9405779 PMID: 36009577.

In summary, our results clearly demonstrate anti-tumor effects of propofol in GSCs and DGSCs. Propofol induced a direct anti-tumor effect on these cells by inhibiting their self-renewal, proliferation, and migration, and by their sensitization to radiation and TMZ. In addition, propofol also inhibited the pro-tumorigenic interaction of GSCs with microglia, therefore impacting both the tumor cells and the tumor microenvironment. Indeed, the interaction of glioma cells and GSCs with the tumor microenvironment plays a critical role in tumor aggressiveness [10]. We identified BDNF-AS as a mediator of propofol effects via decreasing the levels of BDNF, which acts as a glioma cell mitogen and a regulator of microglia functions (Figure 8). Therefore, propofol, which is widely used in GBM surgeries, should be further explored as a potential repurposed therapeutic agent both during resection and as an effective adjunct to radiation and TMZ. The ability of propofol to inhibit the crosstalk of glioma cells with the tumor microenvironment, combined with the lack of cytotoxic effects of propofol on mature neural cells, further emphasizes its importance as a potential anti-tumor agent.

## Data Availability

Not applicable.

## Supplementary Materials

The following supporting information can be downloaded at: https://www.mdpi.com/article/10.3390/cells12151921/s1, Figure S1. Propofol effects on GSCs self-renewal and cell death. GSC-6 (A) and GSC-11 (B) were plated at 100 cells/well in 96-well plates and treated with different propofol concentrations. The number of neurospheres per well was quantified after 14 days and presented as % of normalized control (A,B). Cell death was analyzed in GSC-10 (C) and GSC-2 (E) using LDH assay. Cell death is presented as relative average OD units. Cell apoptosis of propofol-treated GSC-2 was analyzed using caspase 3 activity (D). The results are presented as the means ± SD of three separate experiments. * *p* < 0.05, ** *p* < 0.01, *** *p* < 0.001. Significance was determined by two-tailed unpaired Student’s *t*-test. Figure S2. Propofol does not exert cell death in human neural cells. Human neurons, oligodendrocytes and microglia were cultured with 100 µM propofol for 48 h and cell apoptosis was analyzed using caspase 3 activity (A). Cell death of human neurons, oligodendrocytes, microglia and astrocytes was analyzed by LDH assay (B). The results are presented as the means ± SD of three separate experiments. Significance was determined by two-tailed unpaired Student’s *t*-test. Figure S3. Propofol inhibits migration and enhances the response of DGSC-2 to TMZ. GSC-2 were differentiated in medium consisting of DMEM+10% FCS for a week. DGSC-2 were treated with propofol (20 µM) and cell migration was analyzed using transwell plates with 8 m filter (A). DGSC-2 were treated with propofol alone (10 µM) or with TMZ (25 µM). Cells proliferation was determined by determining cell number via trypan blue exclusion assay (B). Interaction analysis demonstrated a statistically significant interaction between propofol and TMZ that generated a synergistic inhibitory effect on cell proliferation, F(1, 12) = 39.452, *p* < 0.0005). The results represent as the means ± SD of four different experiments. *** *p* < 0.001. Significance was determined by two-tailed unpaired Student’s *t*-test. Figure S4. Silencing of BDNF-As in GSCs. GSC-10 were transfected with a control and two BDNF-AS siRNAs using siMPORTER reagent. The expression of BDNF-AS was determined after 48 h using RT-PCR. The results represent mean values ± SD of three different experiments. *** *p* < 0.001. Significance was determined by two-tailed unpaired Student’s *t*-test. Figure S5. Expression of BDNF in glial tumors. The expression of BDNF-AS was analyzed in primary (A) and recurrent (B) glioma (GBM+LGG) and in primary GBM (C) and LGG (D) MGMT methylated and unmethylated tumors. Kaplan-Meier estimates of overall survival are plotted for GBM patients according to BDNF-AS expression, log-rank P = 0.89 (E). Relative expression of BDNF in LGG (G2, n = 214; G3. N = 225) and GBM (G4, n = 158) was determined according to The Cancer Genome Atlas (TCGA data portal, Wilcoxon *t*-test *p* < 0.0001) (F). BDNF expression was also determined in glioma tumors (GBM and LGG) by IDH status (IDH WT, n = 217; IDHmut-codel n = 256; IDHmut-non-codel, n = 167, Wilcoxon *t*-test *p* < 0.0001) (G), and molecular subtypes (PN, n = 242; NE, n = 100; CL, n = 80; ME, n = 90, Wilcoxon *t*-test *p* < 0.001) (H). The expression of BDNF in primary (n = 144) and recurrent (n-12) LGG tumors, *p* < 0.0001 (I) and primary (n = 425) and recurrent (n-14) GBM tumors (J) was also analyzed. Kaplan-Meier estimates of overall survival are plotted for GBM patients according to BDNF expression, log-rank P = 0.73, (K) and LGG patients according to BDNF expression, log-rank P = 0.89 (L). Figure S6. Propofol effects on extracellular vesicle (EVs) secretion. The effect of GW4869 (20 µM) on EV secretion was analyzed in both GSC-10 and GSC-2 after 20 h of treatment (A). GSC-10 and GSC-2 were treated with propofol 20 µM for 24 h. EVs were isolated using ExoQuick-TC Ultra kit and were analyzed for the relative expression of CD63, CD81 and CD9 using ELISA (B). The results represent mean values ± SD of three different experiments. ** *p* < 0.01, *** *p* < 0.001. Significance was determined by two-tailed unpaired Student’s *t*-test. Table S1. De-identified patient information of GSCs. Table S2. Sequences of primers used for RT-PCR.

## Abbreviations

bFGF	basic fibroblast growth factor;
CTGF	connective tissue growth factor;
EVs	extracellular vesicles;
EGF	epidermal growth factor;
FN	fibronectin;
GBM	glioblastoma;
GSC	glioma stem cell;
LGG	low-grade glioma;
miRNA	microRNA.
