# Supplementary material for: Propofol Inhibits Glioma Stem Cell Growth and Migration and Their Interaction with Microglia via BDNF-AS and Extracellular Vesicles
Source: Cells. 2023 Jul 25;12(15):1921. doi: 10.3390/cells12151921 (PMC10417602; doi:10.3390/cells12151921)
Supplement: Supplementary file 1 [file cells-12-01921-s001.zip › cells-2389609-supplementary.pdf]

Supplementary figure legends

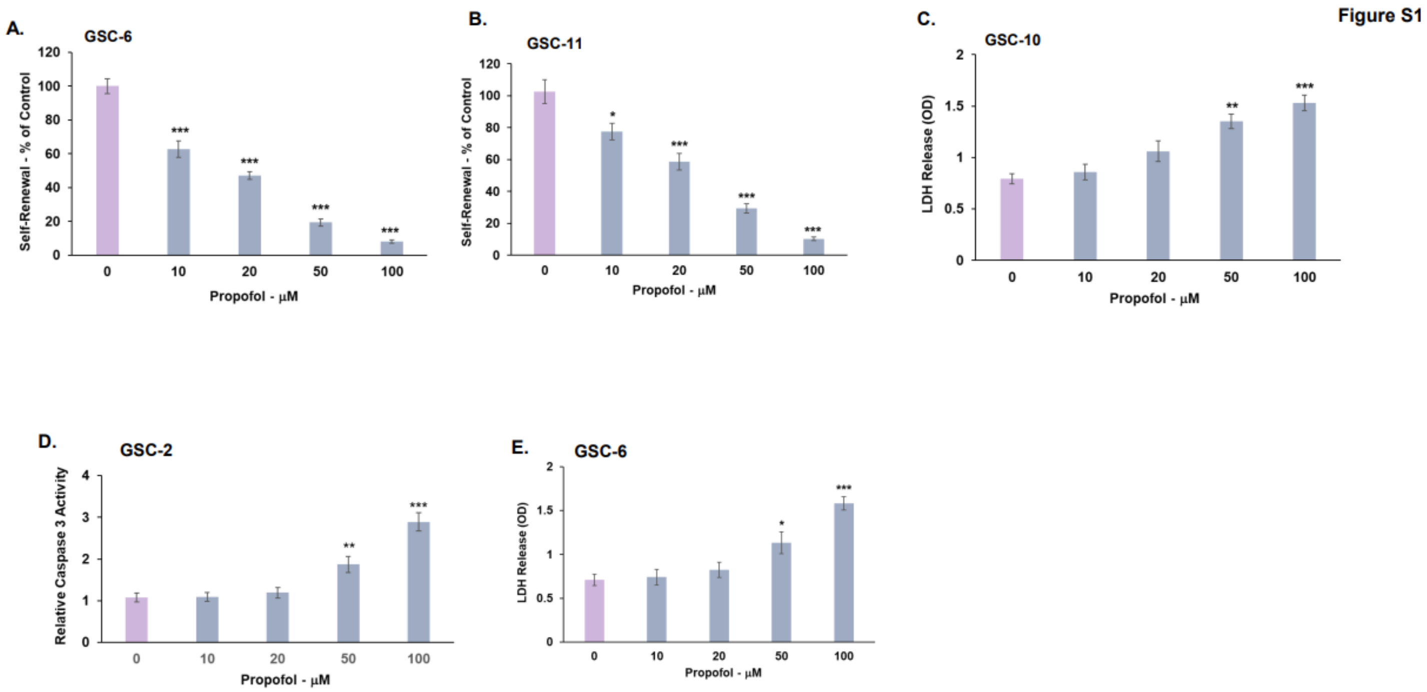

Figure S1. Propofol effects on GSCs self-renewal and cell death. GSC-6 (A) and GSC-11 (B) were plated at 100 cells/well in 96-well plates and treated with different propofol concentrations. The number of neurospheres per well was quantified after 14 days and presented as % of normalized control (A,B). Cell death was analyzed in GSC-10 (C) and GSC-2 (E) using LDH assay. Cell death is presented as relative average OD units. Cell apoptosis of propofol-treated GSC-2 was analyzed using caspase 3 activity (D). The results are presented as the means ± SD of three separate experiments. *P<0.05, **P<0.01, ***P<0.001. Significance was determined by two-tailed unpaired Student's t-test.

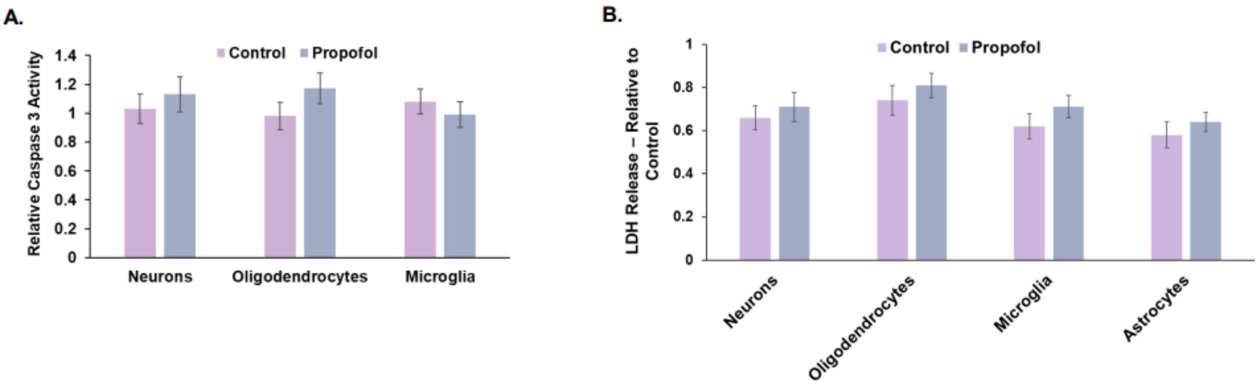

Figure S2. Propofol does not exert cell death in human neural cells. Human neurons, oligodendrocytes and microglia were cultured with 100 μM propofol for 48 hr and cell apoptosis was analyzed using caspase 3 activity (A). Cell death of human neurons, oligodendrocytes, microglia and astrocytes was analyzed by LDH assay (B). The results are presented as the means ± SD of three separate experiments. Significance was determined by two-tailed unpaired Student's t-test.

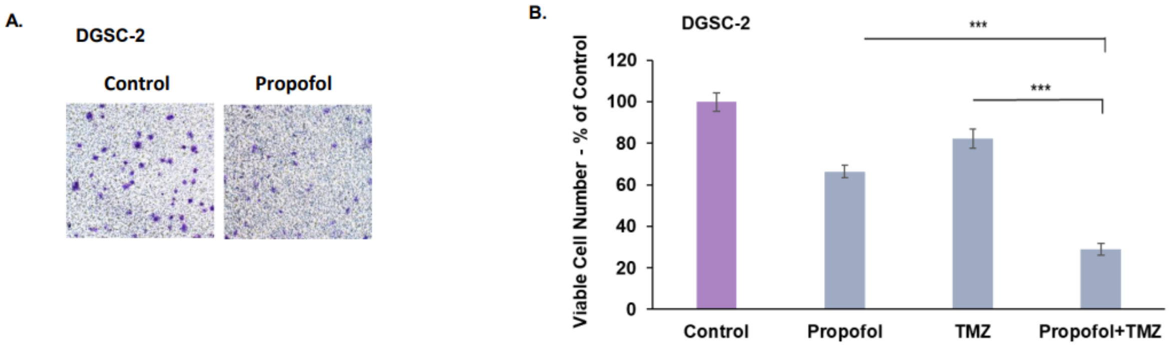

Figure S3. Propofol inhibits migration and enhances the response of DGSC-2 to TMZ. GSC-2 were differentiated in medium consisting of DMEM+10% FCS for a week. DGSC-2 were treated with propofol (20 μM) and cell migration was analyzed using transwell plates with 8 μm filter (A).

DGSC-2 were treated with propofol alone (10 μM) or with TMZ (25 μM). Cells proliferation was determined by determining cell number via trypan blue exclusion assay (B). Interaction analysis demonstrated a statistically significant interaction between propofol and TMZ that generated a synergistic inhibitory effect on cell proliferation, $F(1, 12) = 39.452$, $p < 0.0005$). The results represent as the means ± SD of four different experiments. ***P<0.001. Significance was determined by two-tailed unpaired Student's t-test.

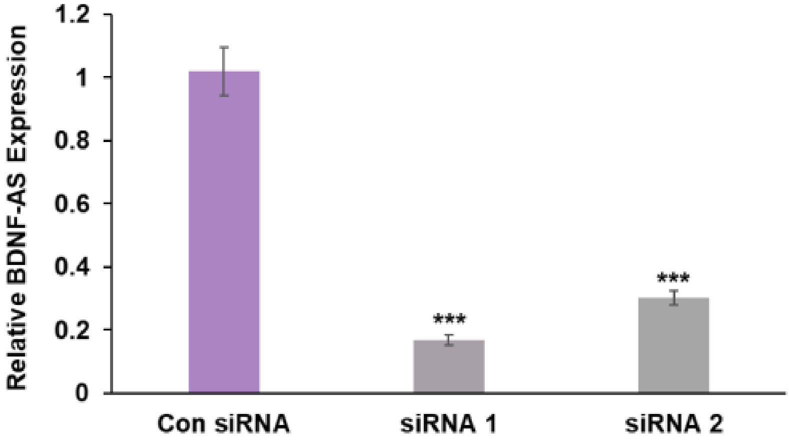

Figure S4. Silencing of BDNF-As in GSCs. GSC-10 were transfected with a control and two BDNF-AS siRNAs using siMPORTER reagent. The expression of BDNF-AS was determined after 48 hr using RT-PCR. The results represent mean values ± SD of three different experiments. ***P<0.001. Significance was determined by two-tailed unpaired Student's t-test.

Figure S5

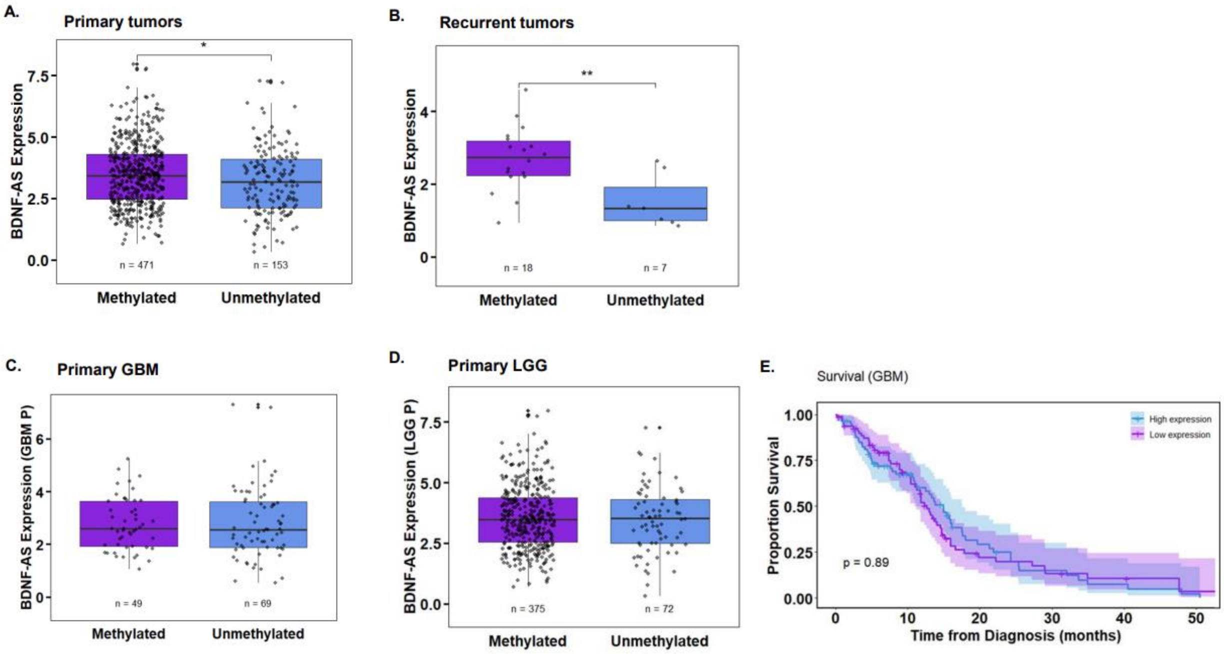

Figure S5

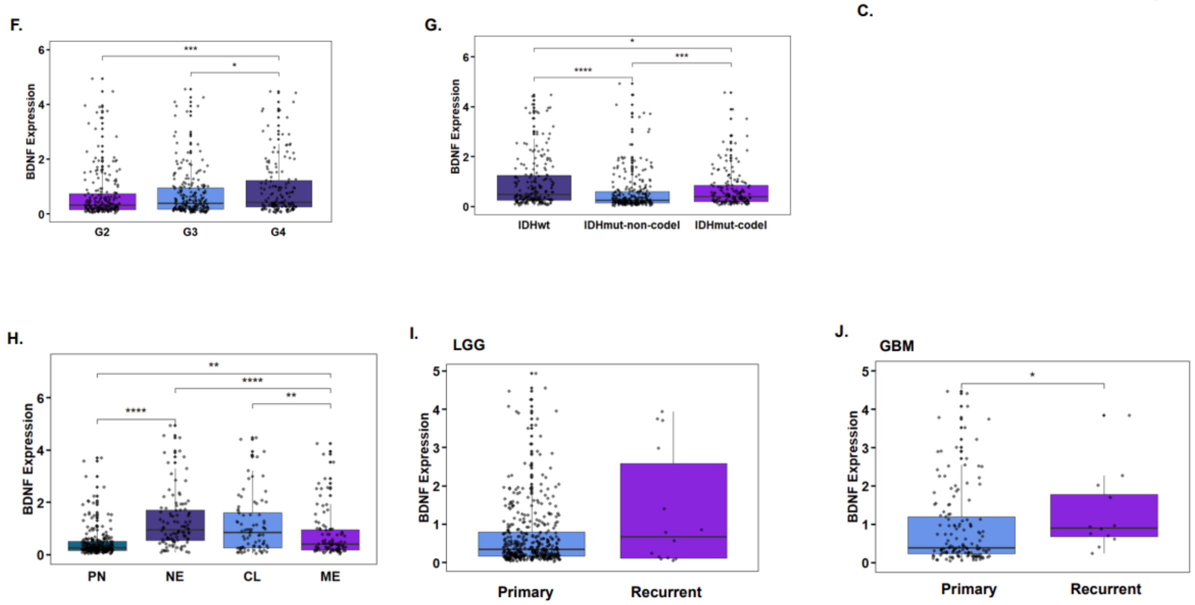

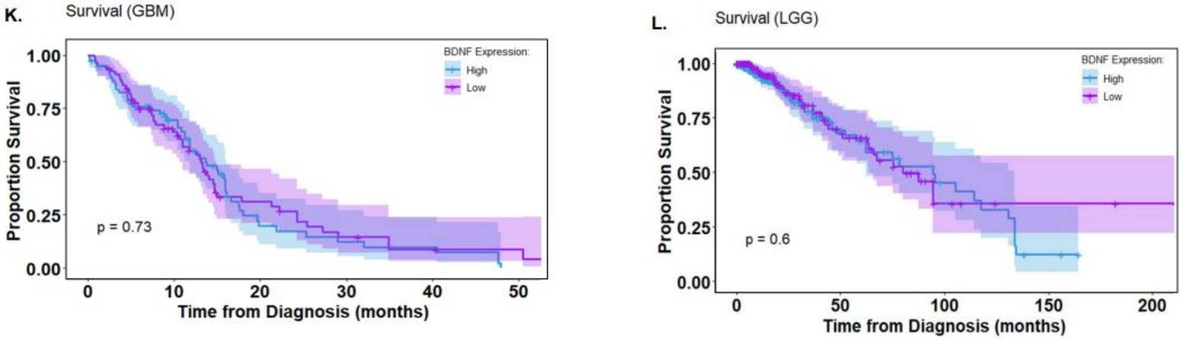

Figure S5. Expression of BDNF in glial tumors. The expression of BDNF-AS was analyzed in primary (A) and recurrent (B) glioma (GBM+LGG) and in primary GBM (C) and LGG (D) MGMT methylated and unmethylated tumors. Kaplan-Meier estimates of overall survival are plotted for GBM patients according to BDNF-AS expression, log-rank P = 0.89 (E). Relative expression of BDNF in LGG (G2, n=214; G3. N=225) and GBM (G4, n=158) was determined according to The Cancer Genome Atlas (TCGA data portal, Wilcoxon t-test P<0.0001) (F). BDNF expression was also determined in glioma tumors (GBM and LGG) by IDH status (IDH WT, n=217; IDHmut-codel n=256; IDHmut-non-codel, n= 167, Wilcoxon t-test P<0.0001) (G), and molecular subtypes (PN, n=242; NE, n=100; CL, n=80; ME, n=90, Wilcoxon t-test P<0.001) (H). The expression of BDNF in primary (n=144) and recurrent (n-12) LGG tumors, P<0.0001 (I) and primary (n=425) and recurrent (n-14) GBM tumors (J) was also analyzed.

Kaplan-Meier estimates of overall survival are plotted for GBM patients according to BDNF expression, log-rank P = 0.73, (K) and LGG patients according to BDNF expression, log-rank P = 0.89 (L).

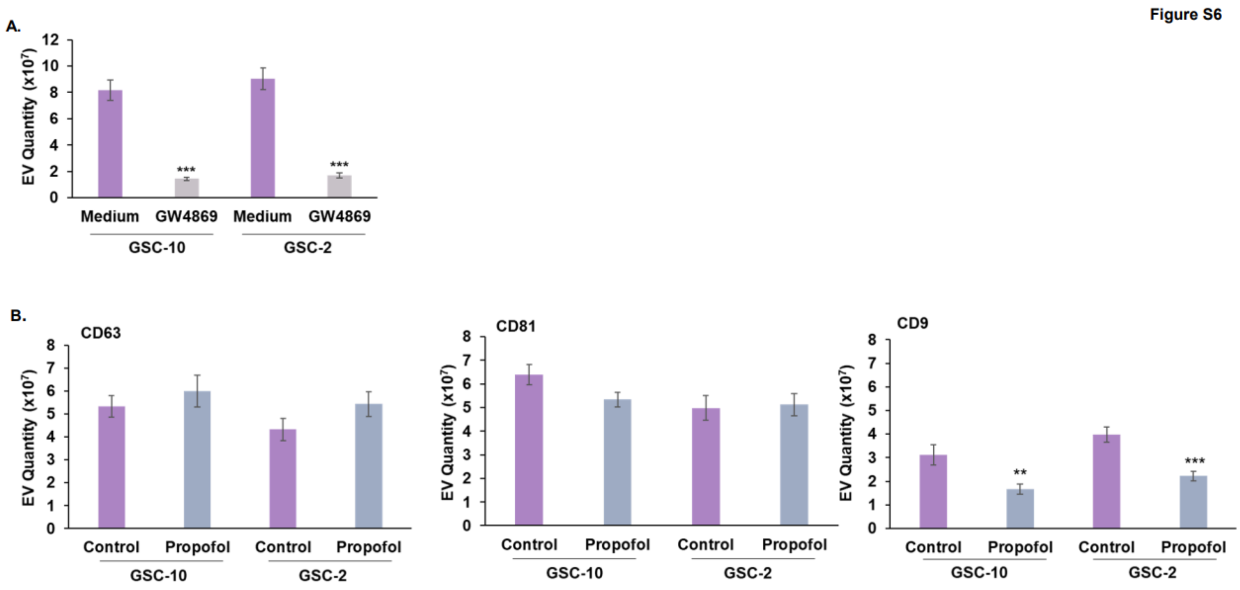

Figure S6. Propofol effects on extracellular vesicle (EVs) secretion. The effect of GW4869 (20 μM) on EV secretion was analyzed in both GSC-10 and GSC-2 after 20 hr of treatment (A). GSC-10 and GSC-2 were treated with propofol 20 μM for 24 hr. EVs were isolated using ExoQuick-TC Ultra kit and were analyzed for the relative expression of CD63, CD81 and CD9 using ELISA (B). The results represent mean values ± SD of three different experiments. $^{**}P<0.01$, $^{***}P<0.001$. Significance was determined by two-tailed unpaired Student's t-test.

Supplementary Table 1. De-identified patient information of GSCs

Cell Line	Age@diagnosis (years)	Gender	OS (days)	MGMT	P53 mutation status	Mesenchymal markers	IDH status
**GSC-10**	39	F	717	M	wt	L	WT
**GSC-2**	54	M	432	M	wt	H	WT
**GSC-6**	54	F	339	U	R175H	H	WT
**GSC-11**	45	M	646	U	M133T	L	WT

For each GSC, the age, gender, survival data, MGMT (U-unmethylated and M-methylated), IDH and p53 status are presented. Mesenchymal phenotypes of the GSCs were determined by the relative expression of YKL40, SMA and GTGF.

Table S2
Sequences of primers used for RT-PCR

**BDNF**	**F:** GACAAGGCAACTTGGCCTAC	**R:** CGTGTTCGAAAGTGTCAGCC
**BDNF-AS**	**F**: CCGTGAGAAGATCTCATTGGG	**R**: CGTGCTCAAAAGTGTCAGCC
**TGF-β1**	**F**: CAAGCAGAGTACACACAGCAT	**R**: TGCTCCACTTTTAACTTGAGCC
**CD44**	**F:** CTCCACCTGAAGAAGATTGT	**R:** AAGATGTAACCTCCTGAAGT
**Vimentin**	**F:** GCAAAGATTCCACTTTGCGT	**R:** GAAATTGCAGGAGGAGATGC
**OCT4**	**F:** ATCAGCCACATCGCCCAGCA	**R**: CCCAGCAGCCTCAAAATCCT
**Nanog**	**F:** ACCTATGCCTGTGATTTGTGG	**R**: GTTGTTTGCCTTTGGGACTG
**SOX2**	**F:** CCAGAAAAACAGCCCGGACC	**R**: CGCTTCTCCGTCTCCGACAA
**YKL40**	**F:** TGCCCTTGACCGCTTCCTCT	**R**: TTGATGAAAGTCCGGCGACT
**CTGF**	**F:** GTGTGCACCGCCAAAGATG	**R**: CAACCACGGTTTGGTCCTTG
**Twist1**	**F:**TAGAAGTCTGAACACTCGTT	**R**: AATTCCTCTGATTGTTACCATT
**IL-10**	**F**:TCTCCGAGATGCCTTCAGCAGA	**R**: TCAGACAAGGCTTGGCAACCCA
**CD86**	**F**: AGCCTTATCGGAAATGATCCAG	**R**: GGCCTTGTAGACACCTTGGT
**CD206**	**F**: CATCAGGGTGCAAGGAAGG	**R**: GTCCAGGCACTGAAAGTGGA
**S12**	**F**:TGCTGGAGGTGTAATGGACG	**R**: CAAGCACACAAAGATGGGCT
**β2MG**	**F:**TAAGTGGGATCGAGACATGTAAGC	**R**: CTAGAGCTACCTGTGGAGCA
